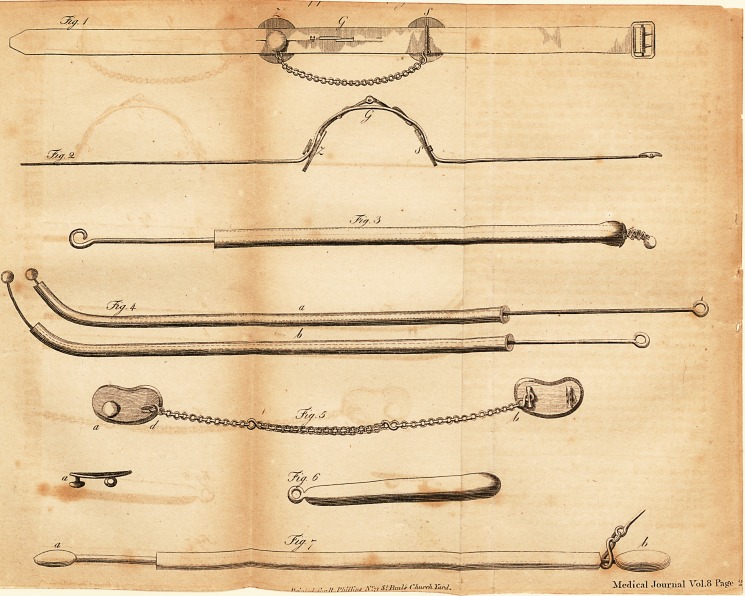# Observations and Experiments, Made with the View of Employing Galvanism for the Cure of Certain Diseases

**Published:** 1802-09-01

**Authors:** 

**Affiliations:** Berlin


### *Observations and Experiments, made with the view of employing Galvanifm for the Cure of certain Diseases; by Dr.* GRAPENGIESSER, *of Berlin.* *

[ With two Plates. ]

IT is feveral years fince I firft conceived the idea of employing Galvanifm in difeafes, having at that time a frequent opportunity of obferving the remarkable effects of the Galvanic ftimulus on the nervous fyftem, as I affifted at the Galvanic experiments of my friend Mr. Humboldt. I fince employed the fimple Galvanifm, or the experiment of Mr. Hunter, (which confifts in touching the different branches of the ramus fecundus paris quinti in the mouth, on the upper jaw, the infraorbitalis and alveolaris, with a piece of zinc and filver, the ends of which are alternately joined and feparated) as a proof for the degree of excitability of the optic nerve. The appearance of light, however, was frequently not perceived, notwithftanding there was a fufficient degree of excitability left in the nerve, and even in the eyes, that were quite found; and though I had often recommended the experiment in weaknefs of fight, I never faw any great fuccefs. In November 1800, a cafe occurred to me, which afforded the *first opportunity of employing the simple Galvanism for the cure of a disease.* A young lady, eighteen years of age, of a lean habit, and pretty tall, had been affected for about four years with a hoarfenefs, which fometimes rendered her quite fpeechlefs, or brought on a perfect aphonia. Having ufed the moft efficacious remedies, as mercurials, cicuta, belladonna, &c. without effect, fhe applied to me for advice. I propofed to lay a blifter round the neck, which, however, produced no change in the difeafe, notwithftanding its being kept in fuppuration for feveral days. I now thought to increafe the effect of the blifter by a Galvanic experiment; and having accordingly placed on each fide of the larynx, a blifter of the fize of a fhilling, I covered the excoriated fpots on one fide with a zinc plate, to which a wire of the fame metal was faftened, and on the other fide with a filver fpatula. As foon as I brought

* From his work, entitled, " *Versuche den Galvanismus zur Heilung einiger Krankheiten anzuwenden, Angestellt und Beschrieben von* C. J. C. Grapengieffer, M. D. *mitz Kupfertfelan, Berlin,* 1801, *for Milius.*" This work being at prefent the beft publication that has appeared on the medical ufe of Galvanifm, we beg leave to communicate its interefting contents more fully than a mere extract would have allowed, for which the novelty and intereft of the fubject will, we hope, make a fufficient apology.

Fig. 1
Z
G
S
Fig. 2
G
Z
S
Fig. 3
Fig. 4
a
b
a
d
Fig. 5
b
a
Fig. 6
a
Fig. 7
b brought the two metals in contact with each other, a burning fenfation at thofe fpots arofe, and the larynx heaved up and down convulfively with loud fobs or fingultus, which were very much increafed whenever I effected the combination of the above metals by means of a gold wire. On alternately applying and removing the wire, the convulfive motions of the larynx became fo violent as almoft to be infupportable to the patient; whereas, when the three metals were in continual contact with each other, the contractions of the mufcles were moderate, and the fenfation lefs troublefome. After I had continued this experiment for about a quarter of an hour, a watery humour began to run from the excoriated furfaces, which, however, proved not fo fharp as had been obferved on fimilar experiments of Mr. Humboldt. The experiment being difcontinued, I dreffed the wounds, after which the convulfive heaving of the larynx by no means ceafed, but recurred from time to time till the evening, on which account I was obliged to prefcribe an opiate, as the family of this lady began to be anxious about her. The patient brought up a great deal of pituitous matter, and two hours after fhe began to fpeak louder and clearer than fhe had hitherto been able to do. This having continued the next day, when the fpaftick contractions of the larynx had entirely ceafed, the fpeech was loft again after the fourth and fifth day. Encouraged, however, by thofe favourable appearances, I determined to repeat the experiment once more, and for a longer time; for which purpofe I procured two metallic plates, one of zinc and the other of filver, which I faftened to a ftrap of leather, in fuch a manner that I could move one of the plates in order to bring them more conveniently in contact with each other by means of a fmall pair of golden pincers, to which end the zinc plate has a button, which is faftened in a hole of the ftrap; the filver plate, however, is furnifhed with a loop fo that it can be moved. The pincers may alfo be faftened by fmall holes in the ftrap. In order to combine thefe two plates more clofely, I frequently place between them a fmall golden chain, See Tab. I. fig. 1, 2. After having again applied two fmall blifters on each fide of the larynx, I faftened the ftrap round the neck, that the plates might exactly lie on the excoriated fpots, and when I brought them in contact by means of the gold pincers, the fame phenomena appeared as were obferved in the firft experiment; they were, however, more intenfe than before, which might be owing to the clofe connexion of the metals produced by this apparatus. Every five, eight, or ten minutes, a convulfive rifing of the larynx came on, attended with a difficulty of deglutition; a great quantity of watery humours iffued from the the furfaces of the wounds, and a great deal of pituitous matter was brought up, whereby the voice became inftantly clearer. On the evening of the fame day, the patient could fpeak perfectly loud, though the quantity of mucus in the throat prevented her from fpeaking diftinctly. The apparatus not being any longer troublefome to her, I did not remove it that night, and though the convulfive heaving of the larynx had from time to time recurred, fhe enjoyed fome reft during the night. The next morning the wounds were healed; and on having removed the apparatus the patient could fpeak pretty loud, though the mucus made the fpeech lefs clear. In order to remove this obftacle I prefcribed an expectorant, and the next day an emetic, which having fufficiently operated, the voice of the patient was as diftinct and loud as it had ever been before*. The fuccefs of this experiment, which I may venture to fay was the firft undertaken with the ferious view of curing a difeafe, had naturally entitled me to entertain the higheft expectations from this remedy. I began to think, how it might be employed in other difeafes, ifchiatic, white fwelling, &c. in which I had already made feveral fuccefsful experiments, when I became acquainted with the difcovery of Volta's pile. A new field of inquiries and experiments opened itfelf, and not a trifling advantage was to be expected from an apparatus, whofe efficacy could be leffened or increafed, and adapted to the incitability of every fubject.

Sect. 2. *Some remarks on Volta's pile and its poles.*—Suppofing

---

* In this favourable ftate fhe remained for about fix months, during which time fhe had once a cold and fore throat, without fuffering any change in her voice, when fhe loft it on a fudden. This difagreeable circumftance feems to be owing to her having expofed herfelf to frequent colds and drafts of air, and fhe was befides very paffionate; at leaft, I could not difcover any other caufe. I tried to produce commotions of the organs of fpeech by means of Volta's pile. To this end I applied two conductors, as fig. 7, Tab. 1, to each fide of the larynx, which were faftened to the two poles of the battery; but though it caufed the fame phenomena as in the above experiment, it produced no effect upon the complaint. I again applied the blifters and the collar, which, at this time, however, had no effect, moft probably on account of the incitability being exhaufted by the ftronger effect of the battery. I determined, therefore, to wait till it was again fufficiently accumulated, during which time I gave pills of *gu. ammon. asafetid.* Sapo Starkeyan, extr. Arnicæ, *Kermes miner. and squill*, and a blifter in the neck was kept open. A fortnight after I began to apply the blifter and the collar, which produced the ufual effect, though in a lefs degree, and feveral hours after the voice was reftored, but the next morning it again entirely difappeared. I fhall hereafter give a farther account of this cafe, as I purpofe to employ the Galvanifm once more at a proper time, after having made the patient undergo a preparative treatment.

posing that the greatest part of our Readers is sufficiently acquainted with the construction of the Galvanic apparatus of Signor Volta, I shall only subjoin a few remarks.—According to my observations, a pile constructed of silver and zinc, acts equally strong as one of gold and zinc, the strength of which, however, is superior to that of a pile of copper and zinc. Having received about a hundred plates of Frederics d'or, not yet struck from the mint at Berlin, I got made an equal number of silver plates; but the piles I constructed with them showed no remarkable difference in their effects; however, I do not deny that some difference may be perceived by means of the Galvanometers proposed by Messrs. Robertson and Simon. For the medical application of Galvanism I am inclined to allow the preference to the copper-plates, on account of the greater uniformity with which they seem to act. The Naturalists of Berlin have agreed, in case the zinc is placed beneath the disc of wet cloth or pasteboard, and the silver above it, to call the undermost end the zinc-side or the zinc-pole, and the uppermost, the silver-side or silver-pole. Mr. Ritter arranged the plates in the following order (beginning from below): Silver, cloth, zinc, silver, cloth, zinc, and so forth. The undermost plates of silver, however, are of no use at all, as it is more than probable, that the action of the two heterogeneous metals merely arises from a process of oxydation; hence a pile constructed of metallic plates only, without any moist body between them, would have no effect at all. If zinc, therefore, is placed beneath the moist intermediate body, and the silver above, the lower end is the zinc-pole, and the upper extremity the silver-pole; the former shews negative and the latter positive electricity. On the decomposition of water, the zinc-pole produces hydrogen, and the latter oxygen gas; the former acts with greater force and more penetrating than the latter: The former occasions an alkaline, and the latter a sourish taste; and the former produces in the eyes a reddish light, the latter a bluish. I place beneath the undermost zinc-plate another of the same metal, which at one side projects beyond the former, as seen in T. II. fig. 3,* and a similar silver or copper plate I put on the uppermost silver or copper-plate of the battery. These two plates I call conducting-plates, and they have the advantage, that by placing the uppermost of them between the plates of the pile, lower down or farther up, I am enabled to use for my experiments any number of strata, without in the least deranging the pile, and thus diminish or increase the strength of the

* This Plate will be given in our next Number.

the battery according to the different purpofes that may be intended. Any other conftruction of the Galvanic battery, which does not allow the removing and adding of ftrata, according to the different intentions, is not well calculated for medical ufe; for inftance, the conftruction of Volta's pile, propofed by Mr. Cruikfhank, (Nicholfon's Journal for Natural Philofophy, Vol. iv. p. 258) by cementing the plates and faftening them in a kind of trough, with intermediate cells, filled with a folution of falt or fal ammonia.

The Galvanic battery is by degrees deprived of its power,

1. When the difcs of cloth or pafteboard are dry, either by the evaporation or decompofition of the fluidity.

2. When all the plates are oxydated at the furfaces towards the difcs. In this cafe the pile muft be taken down, and the plates freed from the oxydation by a file or by rubbing them with fand, falt and vinegar, or very diluted fulphuric acid.

I alfo beg leave to point out fome other advantages in the management of the apparatus.

1. As foon as the battery begins to lofe its power, the firft method of renewing its action, is to fhake the chains in different directions, or, what is ftill better, to remove them from each other, and to ftretch them very tight, which is to be done with dry hands. The power of the battery will be alfo renewed by placing the conducting-plates between fome of the plates of which the pile is conftructed.

2. Not the leaft oxyd ought to be fuffered in the holes of the conducting-plates, or in the links of the chains, as this will prevent its being properly conducted.

3. Of all fubftances that kind of cloth called Karfimir is beft calculated for retaining the moifture, becaufe common cloth becomes ftiff and hard, and pafteboard is not fufficiently durable.

There is, however, a ftate of deafnefs originating from debility with increafed excitability, in which I have never experienced any great fuccefs from the application of Galvanifm. Patients of this kind hear lefs diftinctly when there is much noife and the found ftrong, as from a hearing trumpet; but in order that they may underftand what is faid, it is neceffary to fpeak gently and diftinctly clofe to the ear; they hear better in moift weather, and after good reft. In deafnefs arifing from an accumulation of blood in the veffels of the head, and more efpecially of the organ of hearing, the application of Galvanifm is extremely hurtful; and it is likewife of no effect, where retrograde exanthemata, gout, or rheumatifm have occafioned the deafnefs, particularly when thefe caufes continue to act; but if the difeafe fhould remain after they have been removed, the application application of Galvanifm is properly indicated. The accurate diagnofis of thefe different fpecies of deafnefs is often fubject to many difficulties, as it fometimes happens that two or three of the above caufes may take place at once, or one fupervene to the other, and thus render the ftate of the difeafe more complicated. Deafnefs and difficulty of hearing produced by a deficiency of any of the auditory organs, by external lefion, commotions of the brain, relaxation of the membrana tympani, &c. are not properly calculated for the application of the Galvanic ftimulus. Something remains to be faid of the tinkling of the ears (fufurrus aurium), a fymptom which frequently attends deafnefs, and is generally thought to throw light on the nature of fome fpecies of this complaint; but from my experiments I am induced to contradict this opinion. The tinkling of the ears is either, 1, A particular fymptom, which, without having the leaft influence on hearing, forms a peculiar complaint; or, 2. It arifes from the fame caufe with deafnefs, and confequently is a fymptoma caufae, but never a fymptoma morbi, or pathagnomonicum.

In the *firft* cafe it is often of fhort duration, and is produced by caufes fometimes inexplicable; or in plethoric perfons, from congeftions of blood towards the head, &c. but in other cafes it is occafioned by fome changes in the auditory organs themfelves, viz. a topical debility of the auditory organ, which is generally attended by a congeftio fanguinis paffiva; an exanthematous eruption in the ear, &c. A difficulty of hearing, however, frequently attends thefe complaints, though not in every cafe. The tinkling of the ears alfo fupervenes to fome fpecies of fevers, particularly to the typhus, and it is not unfrequently met with in cafes of apoplexy.

In the *laft* cafe (2) it is generally concomitant with deafnefs, without being, however, a conftant fymptom of every fpecies of this complaint, as it is fometimes not obferved in the moft perfect deafnefs.

The manner in which Galvanifm acts on that curious fymptom, may ferve us as an indication, whether it is properly employed or not. If, for inftance, 1, *In the deafness without susurrus aurium*, this fymptom comes on during the application of Galvanifm, and ceafes as foon as the Galvanifm is difcontinued, we may confider it as a good fign and promifing fuccefs; but if the finging in the ears proceeding from the application of Galvanifm fhould continue for fome time after, the cafe is not fo favourable, though it ftill admits the application of Galvanifm: If, however, that fymptom becomes too violent, with increafed deafnefs, Galvanifm is not at all indicated.

2. *In the deafness attended with susurrus aurium.*—If this fymptom

ſymptom diſappears during the application of Galvaniſm, we may expect the intended ſucceſs from that ſtimulus; but if it has no influence on the ſuſurrus aurium, or produces another kind of ſounding in the ears, which diſappears on the diſcontinuation of Galvaniſm, the ſucceſs of its application is rather doubtful. If it increaſes the ſinging in the ears, together with the deafneſs, in this caſe Galvaniſm is not fit to be applied. It is ſometimes the caſe, that Galvaniſm proves active on hearing without producing the ſuſurrus aurium or changing it, when it attends deafneſs. The kind of ſound perceived in the ſuſurrus aurium is extremely different, as it ſometimes reſembles the hiſſing noiſe of boiling water, or the ringing of bells, the roaring of a ſtorm, &c.

4. *Paralysis of the Spincter Ani and of the Neck of the urinary Bladder.* I am actually engaged in applying the Galvaniſm of the battery againſt the complaint, by bringing the conductor, Tab. I. fig. 6, joined by means of a chain with the zinc-pole, into the inteſtinum rectum, while a ſmall concave ſilver plate is applied to the glans penis and brought in contact with the other pole. The experiment being tried but a few days ago, I cannot yet judge of the ſucceſs.

5. *Asphyxia, or apparent Death.* Galvaniſm may be uſed with great ſucceſs for the diagnoſis of this phenomenon from real Death, and for reſtoring people to life. Having, however, not as yet had an opportunity of making experiments in that Caſe, I refer my Readers to what Mr. Humboldt has ſtated on the mode of applying the ſimple Galvaniſm in this accident; but it ſeems probable, that the battery would prove more efficacious than ſimple Galvaniſm

6. *Chronical Hoarseness and Aphonia.* From the ſtatement of my firſt experiment, it appears how uſeful the application of Galvaniſm may prove in this complaint; but as it is ſometimes occaſioned by different cauſes, on which the action of Galvaniſm cannot operate, it is very material to diſtinguiſh them with accuracy, and to aſcertain the caſes in which Galvaniſm is properly indicated. Hoarſeneſs and aphonia from topical deficiences, preternatural formation of the organs of voice, conſtruction of the larynx and trachea, after inflammations and ſuppuration, cannot be removed by Galvaniſm. It is likewiſe of no effect, but rather hurtful, to apply the Galvaniſm in that ſpecies of hoarſeneſs and aphonia, which ariſes from an inflammation of the wind-pipe and larynx, (angina trachealis et laryngia) or from an increaſed ſecretion of mucus in theſe parts, (blenorrhoea) originating from debility with increaſed irritability, or with increaſed excitement.

There

There are only two Cases, in which Galvanifm is ufeful and efficacious, viz. If the difeafe proceeds,

1. *From indirect Debility and Paralysis of the Nerves of the Voice*; for inftance, after a violent inflammation, after violent efforts of thefe organs by finging, fcreaming, &c. in this cafe the Galvanifm of the battery is to be applied on the wetted fkin, without its being previoufly excoriated by blifters.

2. *From the Action of a morbid Stimulus*, by which a certain change in the organs of fpeech is produced, probably an effufion of lymph between the nervous and mufcular fibres, which hinders and difturbs the function of thofe organs. The nature of that ftimulus is generally catarrhous, rheumatical, gouty, fcrophulous, exanthematous, and venereal, but this is not very material; and if it produces a certain degree of excitment, which Hunter calls adhefive inflammation and a tranfudation of lymph, we may have recourfe to Galvanifm. This ftate partly refembles a fcirrhus, partly a dropfy; at leaft, its tranfition to either of thefe affections may eafily take place, the lymph not being reforbed in due time. Galvanifm, particularly the fimple one, applied on wounds occafioned by blifters, is of the greateft fervice, particularly when the morbific caufes have ceafed to act. General remedies ought, however, not to be entirely neglected in thefe cafes.

7. *The White Swelling*, and 8. *Struma*, in feveral cafes of which I have applied the Galvinifm with fome fuccefs, though my experiments were not numerous nor lafting enough, to enable me to give a more accurate account of the efficacy of this remedy in thofe affections.

9. *Some Species of Chronic Rheumatisms, especially the Sciatica*, the nature of which muft be afthenic, for admitting the application of Galvanifm.

10. I have ufed it with great fuccefs in a topical inflammation, occafioned by a metaftafis of variolous matter.—A child, four years of age, of a weak and delicate conftitution, was afflicted after the fmall-pox with a metaftafis in the bones of the hip and elbow joint, of which laft the child fuffered particularly, as it was fwoln, red, quite immoveable, and extremely painful. Having applied two fmall blifters, one above the joint on the infide of the arm, and the other below the joint on the outfide, I faftened on the wounds two plates, one of filver, the other of zinc; the firft of which was connected by means of a chain with the filver fide, and the other with the zinc fide of a battery of twelve ftrata, and the whole apparatus fuffered to remain in this ftate during the night. On its being removed next morning, the fwelling was found to have confiderably decreafed, the pains were greatly diminifhed, and the child could move the arm in different directions without much pain. The affection in the hip, however, was as bad as before. Having prefcribed calomel, with fulphur auratum antimonii, and a decoction of ftipit. dulcamar. and rad. helenii, I applied the apparatus a fecond time, and though I found it the next morning to be quite deranged, and all the bad fymptoms to have returned, I am induced to expect the intended fuccefs from the continued ufe of this method of cure.

Galvanifm appears to act: 1. As a generally exciting remedy, which, when applied in difeafes of deficient excitement in due time, and in a proper degree, is capable of recruiting life, and reftoring fuch a degree of excitement, as to enable the organs to perform their functions in a proper manner.

2. As a fpecific exciting remedy, which by its pecular irritation or ftimulus, is capable of diminifhing and removing morbid ftimuli. In this manner thofe cafes may be explained, in which it had fuch a fudden, but not permanent effect.

3. As a powerful derivans, when applied to wounds caufed by blifters.

*Sect.* 6. *Methods of applying Galvanism in Diseases.*

1. *In Paralyses of the Extremities.* In thefe affections I have always employed the Galvanifm of the battery in a double manner.

*a.* Only *on the skin, which must be previously wetted,* by touching with two conductors, (as T. 1. fig. 7.) two different fpots of the limb, which are fufficiently provided with nerves; for inftance, when the whole arm was to be galvanifed, the conductors were applied to the region of the arm-pit, or fomewhat lower down, and to the region of the mufculus pronator quadratus. When the fore arm was only to be acted upon, Galvanifm was conducted to the nervus ulnaris, at the end of the elbow joint, or between the wetted fingers. It is to be remarked here, that the conductor of the zinc fide fhould always be applied above the limb on a nervous trunk, while the conductor of the filver fide muft touch the part below, becaufe this is the moft efficacious manner of application. For the purpofe of galvanifing the hand, the conductor of the filver fide, together with the affected hand, muft be immerfed in a bowl of water, while the zinc conductor is applied to the the nervous branches that run above; the veffel into which the hand is put, ought to be as fmall as convenient, becaufe the efficacy of the battery will be too much diminifhed, if the veffel is larger than is requifite. The conductors produce a rednefs on the wetted fkin, and on touching feveral times the fame place with the conductor of the zinc fide, a fmall round hole will appear, which which does not bleed, though an efchar forms itfelf on it by degrees. The heat is likewife increafed in the part which is galvanifed.

*b. On Wounds made by Blisters.* In this cafe we have the double intention,

1. *To produce a strong Irritation* with a fmall number of ftrata. The excitability of a paralytic part is often fo extremely diminifhed, that 150 ftrata are neceffary for producing fome effect; but if a blifter has been applied before on the place, with which the conductor is brought in contact, one-third of the number of ftrata will be only required.

2. *To occasion a Derivation*; for inftance, in paralyfes caufed by chronic rheumatifm, where a tranfudation of lymphatic humours into the vaginal membranes of the mufcles and nerves has taken place. The wounds are, in this cafe, covered with metallic plates, to which the chains are faftened. A great iffue of watery humours, with an increafed heat of the difeafed part, is generally produced by this mode of applying the Galvanic ftimulus.

### EXPLANATION OF TABLE I.

Fig. 1. A fimple Galvanic chain to be applied on wounds made by a blifter. *Z* is the zinc-plate. *S* the filver-plate. *G* the golden pincers, by means of which the two plates are connected. *K* is a golden chain for making the connexion ftill clofer. All is reprefented in half its natural fize.

Fig. 2. The fame in profile without the chain.

Fig. 3. A conductor for the meatus auditorius. The handle of the wire, which paffes through a glafs tube, is covered with filk, leather, or cloth. The crooked end of the wire is fcrewed on, the opening of the glafs tube being too fmall to let it pafs through, which opening is purpofely fo narrow, that the glafs tube may not flip towards the end of the wire.

Fig. 4. A conductor for the tuba Euftachii, agreeing with the former, except the glafs tube being a little bent, and the wire having at its extremity a fpring, with a fmall metallic knob. (*b*) the fame conductor, with a tube lefs bent, in natural fize.

Fig. 5. A fimple Galvanic chain, to be applied behind the ears and on the proceffus maftoidei. *a.* a reniform fomewhat concave filver-plate with a button and a loop. *b*, a fimilar plate of zinc with a double loop. *c*, *b*, a gold chain. *d*, *c*, a filver chain, double at one end, that it may be lengthened. *a*, the filver plate in profile.

Fig. 6. A conductor, made of filver or zinc, for the nofe, mouth, and other parts of the body, in natural fize.

Fig. 7. A conductor, particularly adapted for the eye. It confifts of a ftrong brafs wire that paffes through a glafs tube, on the two ends of which *a*, *b*, an oblong metallic knob is fcrewed

[ To be continued. ]

## Figures and Tables

**Fig. 1 Fig. 2 Fig. 3 Fig. 4 Fig. 5 Fig. 6 Fig. 7 f1:**